# Investigation of acute encephalitis syndrome with implementation of metagenomic next generation sequencing in Nepal

**DOI:** 10.1186/s12879-024-09628-y

**Published:** 2024-07-25

**Authors:** Shrestha Rajeev, Katuwal Nishan, Tamrakar Dipesh, Tato Cristina M, Vanaerschot Manu, Ahyong Vida, Gil Juliana, Madhup Surendra Kumar, Gupta Binod, Jha Runa

**Affiliations:** 1 Center for Infectious Disease Research and Surveillance, Dhulikhel Hospital Kathmandu University Hospital, Dhulikhel, Nepal; 2 Department of Pharmacology, Kathmandu University School of Medical Sciences, Dhulikhel, Nepal; 3 Molecular and Genome Sequencing Research Lab, Dhulikhel Hospital Kathmandu University Hospital, Dhulikhel, Nepal; 4 Department of Community Medicine, Kathmandu University School of Medical Sciences, Dhulikhel, Nepal; 5 Rapid Response Team, Chan Zuckerberg Biohub, San Francisco, USA; 6 Department of Microbiology, Kathmandu University School of Medical Sciences, Dhulikhel, Nepal; 7 Emergency Preparedness and Operation, WHE Program, World Health Organization, Kathmandu, Nepal; 8 National Public Health Laboratory, Kathmandu, Nepal

**Keywords:** Acute encephalitis syndrome, Enterovirus, Human-alphaherpes-virus, Metagenomic next generation sequencing, Nepal

## Abstract

**Background:**

The causative agents of Acute Encephalitis Syndrome remain unknown in 68–75% of the cases. In Nepal, the cases are tested only for Japanese encephalitis, which constitutes only about 15% of the cases. However, there could be several organisms, including vaccine-preventable etiologies that cause acute encephalitis, when identified could direct public health efforts for prevention, including addressing gaps in vaccine coverage.

**Objectives:**

This study employs metagenomic next-generation-sequencing in the investigation of underlying causative etiologies contributing to acute encephalitis syndrome in Nepal.

**Methods:**

In this study, we investigated 90, Japanese-encephalitis-negative, banked cerebrospinal fluid samples that were collected as part of a national surveillance network in 2016 and 2017. Randomization was done to include three age groups (< 5-years; 5-14-years; >15-years). Only some metadata (age and gender) were available. The investigation was performed in two batches which included total nucleic-acid extraction, followed by individual library preparation (DNA and RNA) and sequencing on Illumina iSeq100. The genomic data were interpreted using Chan Zuckerberg-ID and confirmed with polymerase-chain-reaction.

**Results:**

Human-alphaherpes-virus 2 and Enterovirus-B were seen in two samples. These hits were confirmed by qPCR and semi-nested PCR respectively. Most of the other samples were marred by low abundance of pathogen, possible freeze-thaw cycles, lack of process controls and associated clinical metadata.

**Conclusion:**

From this study, two documented causative agents were revealed through metagenomic next-generation-sequencing. Insufficiency of clinical metadata, process controls, low pathogen abundance and absence of standard procedures to collect and store samples in nucleic-acid protectants could have impeded the study and incorporated ambiguity while correlating the identified hits to infection. Therefore, there is need of standardized procedures for sample collection, inclusion of process controls and clinical metadata. Despite challenging conditions, this study highlights the usefulness of mNGS to investigate diseases with unknown etiologies and guide development of adequate clinical-management-algorithms and outbreak investigations in Nepal.

## Background

Acute Encephalitis Syndrome (AES) is defined by acute onset of fever and a change in mental status (including symptoms such as confusion, disorientation, coma, or inability to talk) and/or new onset of seizures (excluding simple febrile seizures) in a person of any age at any time of year [1]. This term was coined by World Health Organization (WHO) in 2008 [1]. Globally, based on various studies, the incidence of AES has ranged from 3.5 to 7.4 per 100,000 patients-years, with a higher incidence among children [2].

The patients suffering from AES usually present acute onset of fever and altered sensorium. This is followed by rapidly worsening clinical conditions and death [3]. The survivors can suffer from long term health issues, including neurological sequelae [3, 4]. The etiologies causing AES can be infectious and non-infectious, with the infectious category comprising of a broad range of organisms (bacteria, virus, parasites) [2, 5]. The causative agents of AES also vary with season and geographic location [6]. Research has shown that the etiologies of AES remain unknown in 68–75% of the cases, while Japanese encephalitis (JE) constitutes about 15% of the cases [7–10]. The landscape of AES, in terms of etiology, has changed in India as well, where outbreak investigations and surveillance studies have increasingly reported non-JEV etiologies [11].

In Nepal, JE is majorly associated with mortality and morbidity among children [12]. Therefore, since 2004, the Ministry of Health and Population of Nepal, supported by the Office of Infection Prevention Division, World Health Organisation (WHO), has integrated JE surveillance with Acute Flaccid Paralysis, Neonatal Tetanus, and Measles in its National Surveillance Network [13]. Until 2011, over 23,000 AES cases were reported by the surveillance network [14]. Due to a lack of knowledge in etiology, AES cases are only tested for JE and clinical management is performed based on this result. The incidence of undiagnosed AES etiology contributes to a high rate of death and morbidity [14]. There could be several etiologies, including vaccine preventable etiologies, that cause acute encephalitis, which upon identification could direct public health efforts for prevention, including expanded use of vaccines or addressing gaps in vaccine coverage. Herpes Simplex Virus (HSV), Varicella-Zoster Virus (VSV), Enterovirus, Adenovirus, and Rubella, as well as emerging pathogens such as Nipah, Chandipura and Chikungunya have all been reported as causative viral agents of AES, while Neisseria meningitidis, Streptococcus pneumoniae, Listeria sp, and Brucella have been reported as causative bacterial agents [2, 15–17].

While molecular methods such as PCR require prior genetic information on causative agents, genomic methods such as metagenomic Next Generation Sequencing (mNGS) can simultaneously identify minute amounts of infections and co-infections of varying origin in a single investigation and assist in the investigation of transmission of such infections [18, 19]. With the recent dramatic decrease in sequencing costs, this technology provides access to genomic information in a scale that can be implemented to fill gaps in routine clinical practice and address epidemiological questions. In addition to identification (identifying genotypes, virulence or pathogenesis), NGS provide information epidemiological investigation (comparative genomics, phylogenetic analysis) [20–23].

This study, employing mNGS to explore the infective etiologies behind AES, complements a growing number of studies that have used a similar approach to investigate encephalitis, including in a Low-and-Middle Income Countries (LMIC) context [16, 24–26]. The identification of such etiologies is an important step in developing effective prevention and treatment measures which in turn will reduce disability and morbidity.

## Methods

### Sample collection and selection

The investigation included a random selection of 90 retrospective cerebrospinal fluid (CSF) samples that were collected by World Health Organization-Immunization Preventable Diseases (WHO-IPD), throughout Nepal, as a part of the National AES Surveillance Network in collaboration with Family Welfare Division (FWD) in 2016 and 2017. The samples are collected from all over Nepal from individuals suffering from AES. These samples had been tested for JE at National Public Health Laboratory (NPHL) and stored at -80^0^C freezer. For this study, only those samples that tested negative for JE were selected. Randomization was done to include three age groups of < 5 years, 5–14 years, and > 15 years. Only some metadata related to the subjects (age and gender) were known. Each sample was provided with unique study codes to maintain privacy.

### Nucleic acid extraction and mNGS

Total Nucleic Acid was extracted from the CSF samples using Zymo Quick-DNA/RNA™ Pathogen MiniPrep (R1042).

The total nucleic acid samples were aliquoted into two sub samples for RNA and DNA library preparation, respectively. The library preparations were done using NEB Library Prep Kit Ultra II RNA for RNA (New England Biolabs, E7770S) library preparation, and NEB Library Prep Kit Ultra II FS DNA for DNA library preparation (New England Biolabs, E7805S). The library preparation for the first 30 samples was done in a single batch (at Chan Zuckerberg Biohub, USA) while the remaining 60 samples were done in three batches of 20 samples each (at Dhulikhel Hospital Kathmandu University Hospital, Nepal). Negative extraction and library preparation controls were included in each batch. The library preparation included 10ng of input nucleic acid, followed by fragmentation, adapter ligation, cleanup (Solid Phase Reversible Immobilization beads), barcoding and amplification of library for 12–16 cycles. With subsequent library preparations, quality control was done using agarose gel electrophoresis and Tapestation 4200 platform from Agilent Technologies and later by qPCR using Kapa Illumina Library Amplification (KK2702) and Quantitation Complete Kit (KK4923). It was made sure that the length of DNA in the libraries was around 350–400 bp and had concentration > 1nM. In RNA library preparation, External Control Controls Consortium, 4,456,740 (ERCC) RNA Spike-in controls were used as internal controls.

The libraries that passed quality control filters were pooled and run on an Illumina iSeq100 sequencer. The sequencing was performed for 2 × 146 bp length using custom unique dual indices of 12 bp length. 5% PhiX was added as an internal control for sequencing. The loading concentration of pooled libraries was maintained at 100-120pM.

### Data analysis

The analysis was performed on the CZ ID (formally known as IDSeq) platform developed by Chan Zuckerberg Initiative and CZ Biohub. CZID accepts raw sequencing data, perform host and quality filteration, followed by execution of assemblybased alignment pipeline [27]. The samples are analysed based on number of reads per million (Number of reads aligning to the taxon in the NCBI NR/NT database, per million reads sequenced), reads (Number of reads aligning to the taxon in the NCBI NT/NR database), contig number (Number of assembled contigs aligning to the taxon in the NCBI NT/NR database), id% and z-score. The samples were also visualized using a rpm heatmap where samples and controls are cross-matched against each other. Respective background models were created, from negative extraction and library preparation control, for RNA and DNA Libraries.

### PCR confirmation

Human alphaherpes virus confirmation was done by qPCR (KAPA HiFi HotStart Ready Mix) using two primer sets: established primers (FP: 5’TGCAGTTTACGTATAACCACATACAGC 3’ and RP: 5’ AGCTGCGGGCCTCGTT 3’) and self-designed primers (FP: 5’ GACTCAAACACGTGCACCAC and RP: 5’ CCATCGCGTACAGCCTACAT 3’) [28]. The primer sets were designed using NCBI primer blast and Gene Script, then checked with Beacon Designer Free and Snap Gene Viewer.

Similarly, for confirmation of Enterovirus, modified protocol with established primers from Enterovirus Surveillance Guidelines were used to perform semi-nested Polymerase Chain Reaction (snPCR) [29]. The protocol followed visualization of the bands, for confirmation, in agarose (1.5%) gel electrophoresis.

## Results

### Subject metadata

The samples selected for this study were banked, retrospective CSF samples collected in 2016 and 2017 with limited metadata such as age and gender. Out of the 90 subjects, 31 (34.4%) were female and the age distribution has been presented in Table 1.

**Table 1:** Age distribution between subjects with AES

SN	Age Group	Number
1	< 5 years	23
2	5–14 years	14
3	> 15 years	53

In this study, the subject AES_28 and AES_47 were 24 years female and 25 years female respectively. The median age of the subjects infected with AES was 20 years (IQR: 4–79 years)

### Sample collection

The samples were collected in glass bottles without any preservative and transported to NPHL where they, first, had been tested for JE and subsequently stored at -80^o^C. As these samples were collected in 2016 and 2017 and banked, negative controls were not available during collection and transportation.

### Nucleic acid extraction and mNGS

The extracted nucleic acid had a concentration ranging from too low to detect to 222 ng/ul. As the analysis was done in two sets. Each set was processed for DNA and RNA Library Preparation and has been presented accordingly. Out of 90 samples, only two samples showed confirmed hits from Enterovirus B and Human-alphaherpes-virus 2, respectively. However, these two targets were not evaluated in other samples due to unavailability of sample volume.

### mNGS of RNA libraries

The results from RNA libraries showed some distinct organisms hit in CZID, and also provided a broad picture of the landscape of taxa across the samples. The following are heatmaps generated from through RNA library preparation.

**Fig. 1:**
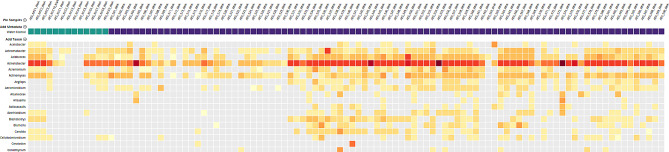
Heatmap depicting top hits from sequencing of RNA library for S01 to S90. The organisms (at the genus level) that were seen in the samples are shown on the left. The names of the samples are on the top of the heat map. The samples marked by green are water controls during extraction (NEC) and library preparation (NLC). The heatmap was generated using the threshold of NT rPM (nucleotide reads per million) > = 10 and NT L (alignment length in base pairs: length of the aligned sequence) > = 50

**Fig. 2:**
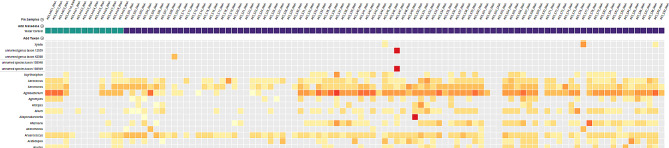
Heatmap depicting hits for Enterovirus (unnamed genus taxon 12,059 and 138,949) from sequencing of AES_S47_RNA. The names of the samples are on the top of the heat map. The samples marked by green are water controls during extraction (NEC) and library preparation (NLC). The heatmap was generated using the threshold of NT rPM (nucleotide reads per million) > = 10 and NT L (alignment length in base pairs: length of the aligned sequence) > = 50

In Figs. 1 and 2, we can see top hits of organisms in the heat map that shows various organisms which are seen at similar levels in the water controls as well. Nevertheless, Pseudomonas genus is seen in all of the samples including few negative controls. There was similar trend, in both sets, with other organism such as Sphingomonas, Acinetobacter, Escherichia and others.

**Fig. 3:**
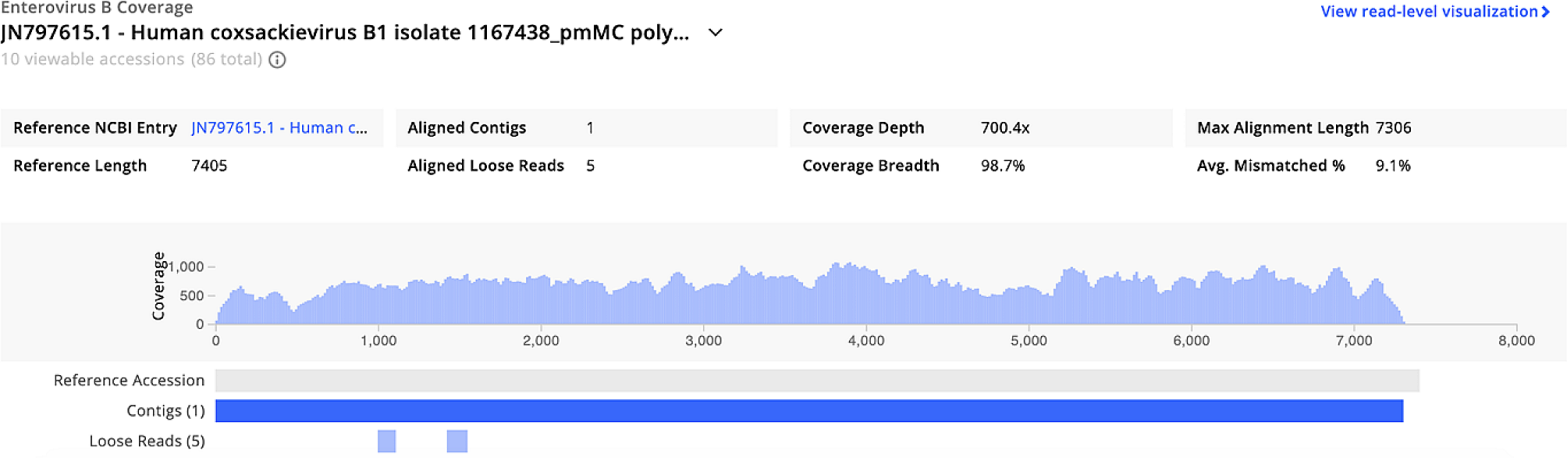
Coverage visualization of Enterovirus in AES_S47_RNA from CZID, depicting the coverage metrics of the contigs generated for the particular hit along with coverage depth and width. The hit was visualized with the threshold of NT rPM > = 10 and NT L > = 50

Interestingly, only AES_S47_RNA showed a hit to Enterovirus B (strain Human coxsackievirus B1). This hit was particular to sample 47 and not seen in any negative controls. The metrics such as rPM of 359,409.1 (provides information of the abundance of a specific microbe within the sample), NT L (depicts the length of aligned sequence in base pair), Z score of 99 (shows the significance of any hit compared to the background), coverage visualization (assess breadth and depths of reads) and id of 85.8% signify that the organism hit is highly similar to the reference organism [30]. The figure below shows the abundance of Enterovirus B in Sample 47 (NT rPM > = 10 and NT L > = 50). The coverage breadth of this hit was 98.7% with depth of 700.4x as seen in Fig. 3.

**Fig. 4:**
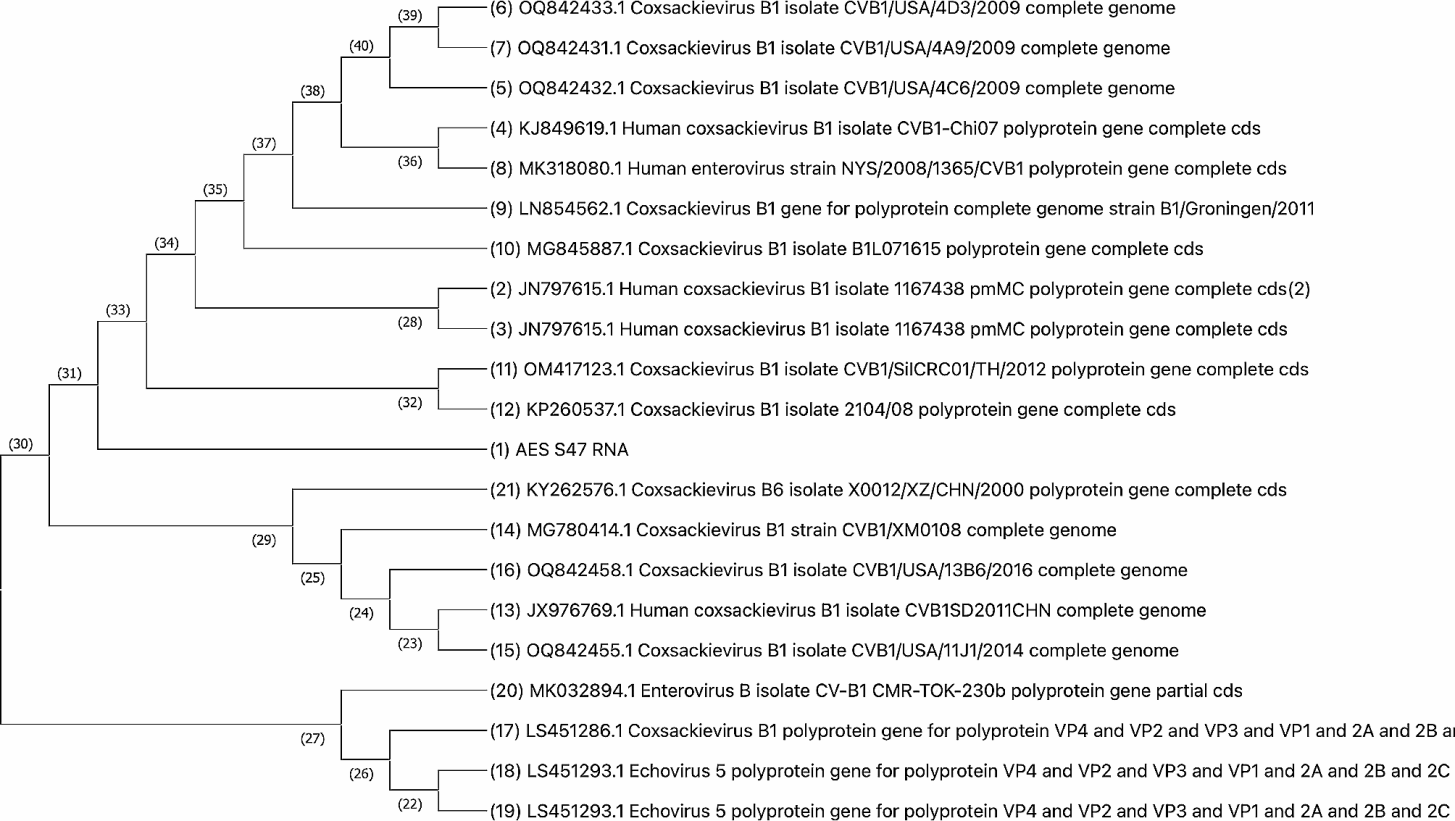
Phylogenetic comparison of AES_S47_RNA against other coxsackievirus B1 genomes, from NCBI. This tree was made using CLUSTAL W maximum likelihood statistical method, Tamura-Nei model with nearest neighbor interchange as the maximum likelihood heuristic method

The strain Human coxsackievirus B1, from our study, was found similar to Coxsackievirus B1 responsible in mesangial renal disease [30]. The genome similarly was also observed in genomes from coxsackievirus viruses causing myocarditis, severe gastroenteritis, food-and-mouth disease, respiratory distress, shown in Fig. 4 [31–35].

**Fig. 5:**
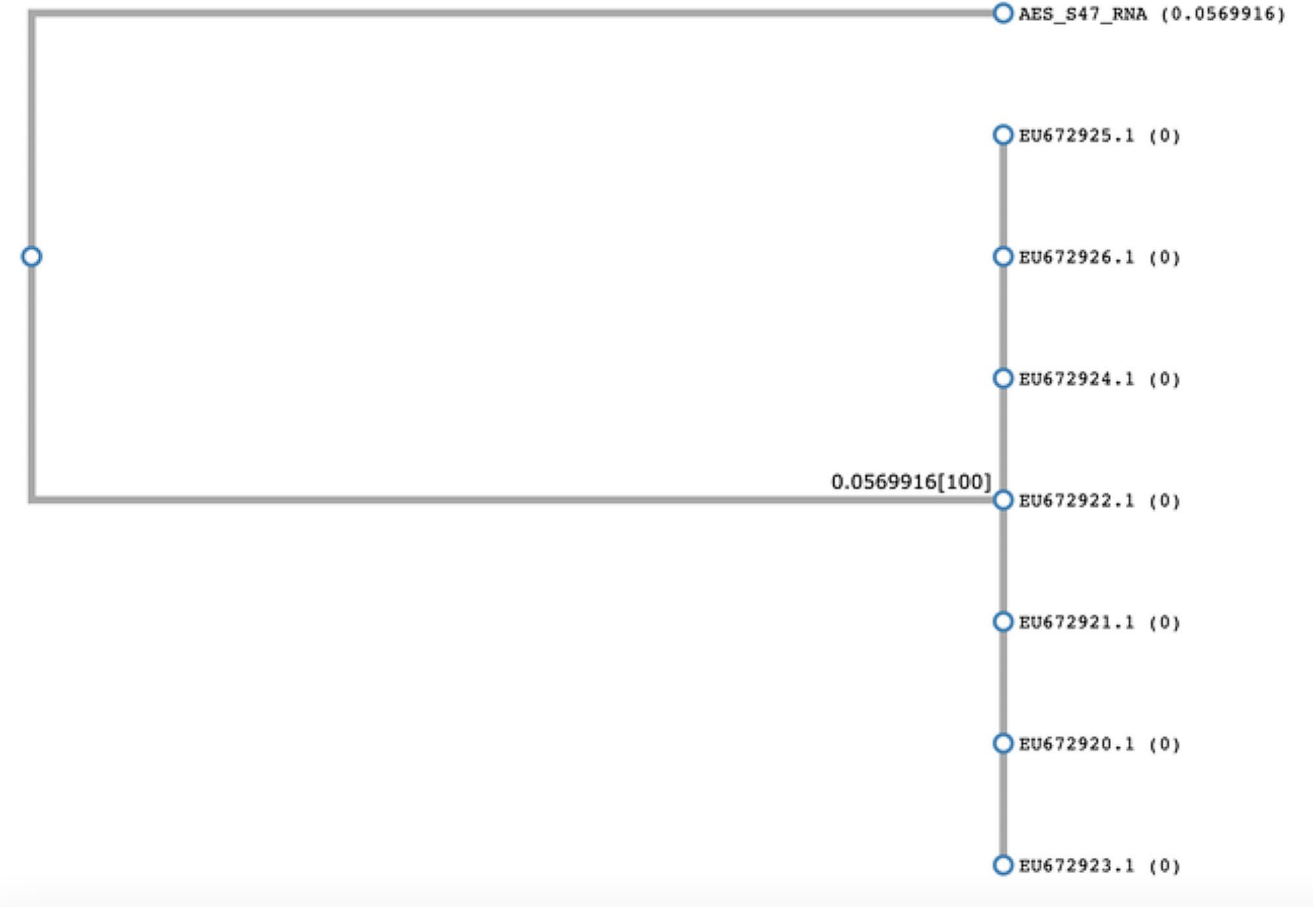
Phylogenic comparison of AES_S47_RNA against coxsackievirus B genomes isolated from an outbreak in India, based on based on partial 5’ noncoding region sequences. This tree was made using CLUSTALW.

The strain Human coxsackievirus B1, from our study, was different (0.0569) from Enteroviruses B isolated from an outbreak in norther India, close in Nepal [62] (Fig. 5).

### mNGS of DNA libraries

**Fig. 6:**
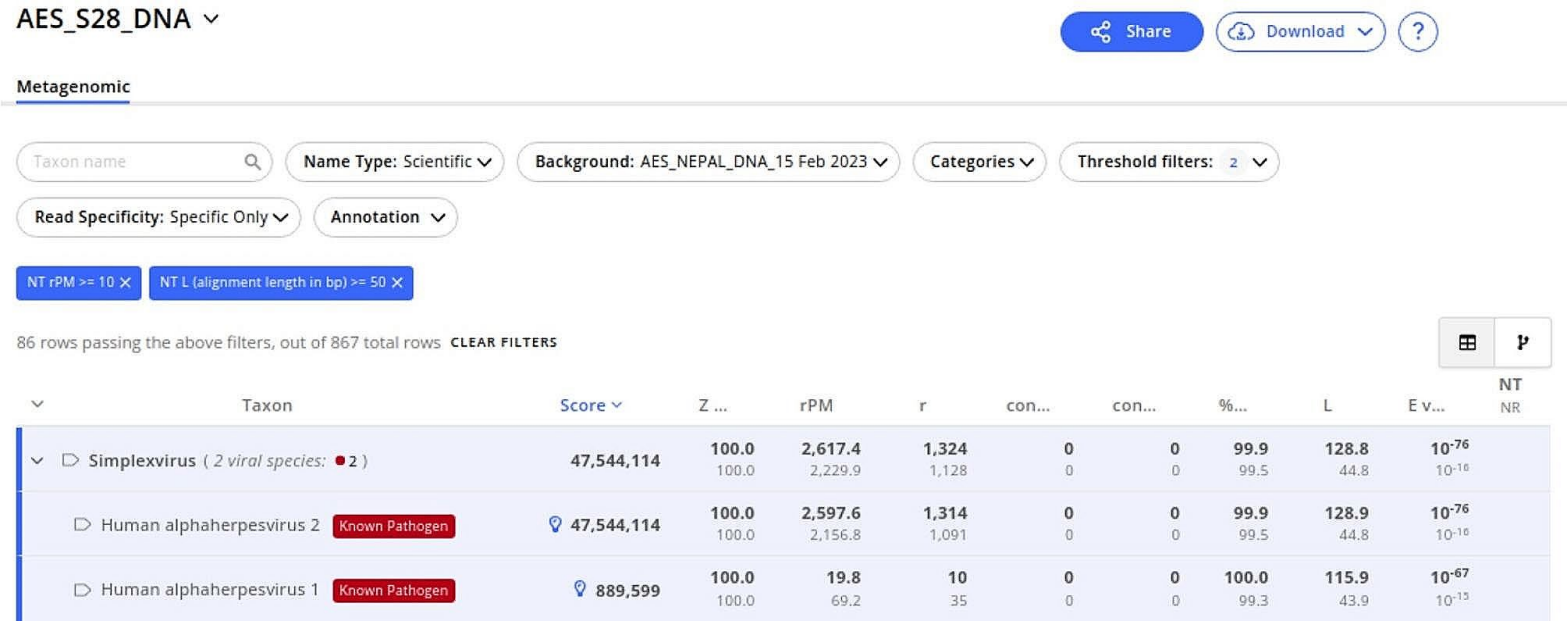
Result from CZID showing the details of top hits in AES_S28_DNA along with various metrics related to the hit. The hits were visualised with the threshold of NT rPM > = 10 and NT L > = 50

In mNGS of DNA libraries, hits were observed for Human alphaherpesvirus 2 [AES_S28_DNA] from the first set. The same sample showed hit for Human- alphaherpes-virus 1, but in a very low abundance, shown in Fig. 4. Additionally, background contaminants (laboratory and hospital) were seen in the water controls in this DNA sequencing result as well. Similar to RNA Libraries, most of the samples showed hits for Sphingomonas spp, Pseudomonas spp, and Acinetobacter spp. Nevertheless, the Fig. 6 shows the result of the hit where there were 2,597.6 rPM for Human-alphaherpes-virus 2.

However, due to lower coverage, contig visualization was not available for this hit.

### PCR confirmation

#### Confirmation of human alphaherpesvirus 2

Out of the two primer sets used, the established primers fared better providing Ct value of 23.11 for Human alphaherpesvirus 2.

### Confirmation of Enterovirus B

After completion of snPCR for Enterovirus, the band was seen between 700 and 800 bp after first amplification and between 300 and 400 bp after final amplification. This confirmed the presence of Enterovirus as per the Enterovirus Surveillance Guidelines [36].

## Discussion

### Demography of acute encephalitis syndrome (AES)

Most of the subjects suffering from AES were young male population of median age 20 years. This gender distribution was concurrent to previous studies done, in Nepal, on epidemiology of AES [37, 38]. It has been observed that AES affects individuals from both gender and all ages, however, most of the studies have been done in younger population, as they pose high risk due to lack of developed antibodies [39–42]. Another study done in Nepal also observed the young median age (19 years) for AES, while others observed older population [37, 38, 43, 44].

### Metagenomic next generation sequencing

In this study, out of the 90 samples tested, most (*n* = 88) of them could not be identified as specific hits. This was due to high level of background contaminants resulting in low confidence in calling organism hits within the experimental samples. Nevertheless, two samples showed confirmed hits for Enterovirus B and Human-alphaherpes-virus 2, respectively, which is in contrast to the studies showing that non-JE pathogens constitutes of 68–75% of AES cases [7–10]. Nonetheless, the absence of causative agent in remaining samples could indicate that either the samples did not have intact nucleic to start with or had low pathogen abundance or could have been degraded because the ERCCs were amply sequenced from the RNA libraries [16, 45].

As per the result of mNGS, the high Z score (99) for Enterovirus B shows that hit for the organism is present significantly in our sample, when compared to the background. The average length of alignment (as shown by L metrics) is long (L = 7258.7), which confirms for a good local alignment to reference [46]. The id% is also higher (85.8%) meaning that the organism is highly similar to the reference organism in the database. Additionally, when the genome coverage is seen in detail, we can see that our sequenced genome depicts good coverage breadth and depth (depth of 700x and breadth of 98.7%), which is the range and uniformity of sequencing coverage for the particular hit [45]. The presence of ENVB was also confirmed through snPCR followed by visualization of product size specific for all enteroviruses [36].

The hit for human-alphaherpes-virus 2 was considered significant because it was not present in the control samples at the thresholds used to analyze the sample (high Z score of 100%, L value of 128.9, id% of 99.9%) considered reliable [24, 27, 47]. The low contig value, for this hit, could be because of the organism being present at such a low abundance that the sequencer did not sequence enough reads to generate a contig. The contig value is dependent upon the total number of reads and the size of organism’s genome [48]. Additionally, the decreased sensitivity of mNGS due to low abundance of pathogen has been studied for CSF [49]. Several methods have been reported that can be used to increase the abundance of pathogen sequences or remove the unwanted host sequences [50, 51]. Nevertheless, as this genus is associated with encephalitis, the sample was taken further for analysis [52, 53]. During confirmation, the lower Ct value of 23.11 indicates presence of human-alphaherpes-virus 2, a known causative agent, in the sample.

### Enterovirus B and Human-alphaherpes-virus 2

Enterovirus B is a known causative agent of encephalitis [16, 54–56]. Enteroviruses are named by their transmission-route through the intestine [57]. Studies have shown that enterovirus can cause various diseases in the nervous system, including aseptic meningitis, acute paralysis, encephalitis, meningo-encephalomyelitis among others, in children [58–60]. Additionally, strain B1 has been documented to cause encephalomyocarditis (meningoencephalitis and severe myocarditis, often accompanied by heart failure) and showed genomic similarity to the enterovirus B from our study [61]. Interestingly, studies in India have linked Enterovirus, among other pathogens, to AES, by various studies [62–64]. Enterovirus outbreak was first reported from Uttar Pradesh, India in 2006 with seasonal outbreaks with high fatality occurring for several years [62, 65, 66]. Southern Nepal borders with Uttar Pradesh, India and due to open borders with similar climate, it is plausible to find Enterovirus in CSF samples in Nepal. However, the strain of Enterovirus from our study was significantly different compared to genomes from the outbreak [62]. Additionally, some studies in Nepal have reported Enterovirus as possible etiology of AES for Nepal [67, 68].

Similarly, Human-alphaherpes-virus 2 is known to cause encephalitis in neonates and immunocompromised patients. Herpes simplex encephalitis (HSE) has significant morbidity and mortality, even with early diagnosis and treatment [69, 70]. HSV is found to be one of the predominant causes of AES in the western world [71–73]. Among HSE, the vast majority of the encephalitis is caused by HSV-1, with HSV-2 being the etiology in less than 10% of the cases [70]. Studies in India and Nepal have reported the presence of HSV-2 as causative agent of encephalitis, with varying range of incidence [69, 74–78].

We believe identification of additional etiologies of AES apart from JEV, which is HSV2 and Enterovirus B from our study, pushes for development for inclusive testing strategies. For instance, qPCR testing could be done for HSV2 and EnvB based on designed primers/probes or commercially available qPCR kits.

### Clinical data and process control

However, due to lack of clinical metadata, the presence of Enterovirus B and Human-alphaherpes-virus 2 could not be clinically correlated. Clinical metadata such as onset of fever, date of infection, fatality, WBC counts, treatment regimes, adjoining infection, etc. are vital to correspond with the presence of infections [16, 79, 80].

Additionally, usual environmental contaminants such as Sphingomonas spp., Pseudomonas spp, or Acinetobacter spp were seen. Sphingomonas species are widely distributed in nature and have been isolated from various land and water habitats, as well as from plant root systems, clinical specimens, and other sources. This is essentially due to their ability to survive in low concentrations of nutrients [81, 82]. Background contaminants of laboratory and hospital origin were also seen in the water controls. With appropriate use of background or negative controls, a background model can be created and subsequently subtracted from the results [16, 24].

### Collection procedures

The lack of identification of causative agent in other 88 samples could be because all of samples that were analysed were as old as 2016 and 2017, and could possibly have gone through numerous freeze and thaw cycles. Therefore, the collection of samples in nucleic acid protectant such as Zymo RNA/DNA Shield would have protected the nucleic acid from degradation after sampling [83, 84]. Additionally, the causative agents could also have left the cerebrospinal fluid prior to collection depending upon the time of collection since the onset of fever, because it is advised to collect CSF within seven days of onset of fever [85].

The possibility of freeze thaw cycles affecting the sample quality and lack of clinical metadata are limiting to the analysis, resulting in ambiguous interpretation of some samples. However, we contend that this aspect should not be corroborated as limitations, because the CSF samples analysed were not collected specifically for mNGS and there could be low abundance of the pathogen itself. Additionally, the sequencing was done in Illumina iSeq100 which has a maximum of approximately 4 million reads per run and can only accommodate a certain number of organisms with adequate coverage breadth and depth [86]. Therefore, more deeper sequencing using sequencer with higher reads per run, host depletion and pathogen enrichment methods can be applied for samples with low pathogen abundance [50, 51].

### Impact of the study

The identification of causative etiologies behind Acute Encephalitis Syndrome (AES) are crucial for developing clinical management algorithms, enhancing surveillance, and formulating treatment and prevention policies. Through this study, we advocate for the utilization of unbiased mNGS to explore the etiologies of under-investigated and undiagnosed febrile illnesses. We do not anticipate the routine use of mNGS as a standard diagnostic tool, but recommended to be a valuable investigational and exploratory instrument for identifying causative etiologies and developing molecular diagnostic methods, such as qPCR.

## Conclusion

Identification and investigation of etiologies behind AES is essential for developing clinical management algorithms, improving surveillance with region-specific treatment and prevention policy as well as outbreak investigation. We do not expect the adaptation of mNGS as a regular diagnostic tool but rather an investigational and exploration tool to identify causative etiologies and develop molecular methods (such as qPCR) for diagnosis. Thus, this study advocates for utlisation of unbiased mNGS to investigate etiologies of under-investigated and undiagnosed febrile illness.

From this study, two documented, causative agents were revealed through metagenomic next generation sequencing and subsequently confirmed by PCR. Insufficiency of clinical metadata, process controls, and possibility of freeze thaw cycles affecting the sample quality incorporates ambiguity when correlating identified pathogens to infections. Therefore, there is a dire need of implementing standardized collection and storage procedures, including proper process controls and clinical metadata (WBC Count, primary diagnosis, discharge type, presence of another organism). Additionally, it is recommended that CSF sample should be collected in a protectant and transported in a controlled and sterile environment.

## Data Availability

The pathogen genomic data can be found in Sequence Read Archive, National Center for Biotechnology Information (NCBI), under BioProject no PRJNA1019500.

## Abbreviations

DNA: Deoxyribonucleic Acid
RNA: Ribonucleic Acid
PCR: Polymerase Chain Reaction
AES: Acute Encephalitis Syndrome
WHO: World Health Organization
JEV: Japanese Encephalitis Virus
HSV: Herpes Simplex Virus
VSV: Varicella-Zoster Virus
mNGS: Metagenomic Next Generation Sequencing
NGS: Next Generation Sequencing
LMIC: Low- and Middle-Income Countries
CSF: Cerebrospinal Fluid
WHO-IPD: World Health Organization Immunization Preventable Diseases
FWD: Family Welfare Division
NPHL: National Public Health Laboratory
NEB: New England Biolabs
ERCC: External Control Controls Consortium
CZ ID: Chan Zuckerberg ID
FP: Forward Primer
RP: Reverse Primer
snPCR: Semi nested Polymerase Chain Reaction
NEC: Negative Extraction Control
NLC: Negative Library Control
NT: Nucleotide
rPM: Reads per Million
NT L: Nucleotide Length
Ct: Cycle of Threshold
HSE: Herpes Simplex Encephalitis

## References

[1] Guidelines for Surveillance of Acute Encephalitis Syndrome (With Special Reference to Japanese Encephalitis) NVBDCP. 2006. Accessed: 18 July 2022. http://www.nvbdcp.gov.in/Doc/AES%20guidelines.pdf.

[2] Granerod J, Crowcroft NS. The epidemiology of acute encephalitis. Neuropsychol Rehabil. 2007;17:406–28. 17676528 10.1080/09602010600989620

[3] Narain JP, Dhariwal AC, MacIntyre CR. Acute encephalitis in India: an unfolding tragedy. Indian J Med Res. 2017;145:584–7. 28948947 10.4103/ijmr.IJMR_409_17 PMC5644291

[4] Srivastava N, Deval H, Mittal M, Kant R, Bondre VP. The outbreaks of Acute Encephalitis Syndrome in Uttar Pradesh, India (1978–2020) and its effective management: a remarkable Public Health Success Story. Front Public Health. 2022;9:793268. 35223759 10.3389/fpubh.2021.793268 PMC8863615

[5] Tripathy SK, Mishra P, Dwibedi B, Priyadarshini L, Das RR. Clinico-epidemiological study of viral acute encephalitis syndrome cases and comparison to nonviral cases in children from Eastern India. J Glob Infect Dis. 2019 Jan-Mar;11(1):7–12. 10.4103/jgid.jgid_26_18 PMC6380098 30814829

[6] National Health Mission. Routine Immunization, Government of India. http://www.nrhm.gov.in/nrhmcomponents/rmncha/immunization/background.html.

[7] AES/JE Cases and Deaths in the Country. National Vector Borne Disease Control Programme. Directorate General of Health Services. Ministry of Health and Family Welfare, Government of India; 2012.

[8] Director. Child Health Division. Teku, Kathmandu, Nepal: Department of Health Services, Ministry of Health and Population. Acute Encephalitis Syndrome/Japanese Encephalitis Data of Nepal; 2012.

[9] Cizman M, Jazbec J. Etiology of acute encephalitis in childhood in Slovenia. Pediatr Infect Dis J. 1993;12:903–8. 8265278 10.1097/00006454-199311000-00002

[10] Potharaju NR. Incidence rate of Acute Encephalitis Syndrome without Specific Treatment in India and Nepal. Indian J Community Med 2012 Oct 37(4):240–51. 10.4103/0970-0218.103473 PMC3531018 23293439

[11] Joshi R, Kalantari SP, Reingold A, et al. Changing landscape of acute encephalitis syndrome in India:a systematic review. Natl Med J India. 2012;25:212–20. 23278779

[12] Rayamajhi A, Singh R, Prasad R, Khanal B, Singhi S. Study of Japanese encephalitis and other viral encephalitis in Nepali children. Pediatr Int. 2007;49(6):978–84. 18045307 10.1111/j.1442-200X.2007.02495.x

[13] Pant GR. A serological survey of pigs, horses, and ducks in Nepal for evidence of infection with Japanese encephalitis virus. Ann N Y Acad Sci. 2006;1081:124–9. 17135501 10.1196/annals.1373.013

[14] Rayamajhi A, Ansari I, Ledger E, Bista KP, Impoinvil DE, Nightingale S, Kumar R, Mahaseth C, Solomon T, Griffiths MJ. Clinical and prognostic features among children with acute encephalitis syndrome in Nepal; a retrospective study. BMC Infect Dis. 2011;11:294. 22035278 10.1186/1471-2334-11-294 PMC3219745

[15] Hsu VP, Hossein MJ, Parashar UD, Ali MM, Ksiazek TG, Kuzmin I, Niezgoda N, Rupprecht C, Bresee J, Breiman RF. Nipah virus encephalitis reemergence, Bangladesh. Emerg Infect Dis. 2004;10(12):2082–7. 15663842 10.3201/eid1012.040701 PMC3323384

[16] Saha S, Ramesh A, Kalantar K, Malaker R, Hasanuzzaman M, Khan LM, Mayday MY, Sajib MSI, Li LM, Langelier C, Rahman H, Crawford ED, Tato CM, Islam M, Juan YF, de Bourcy C, Dimitrov B, Wang J, Tang J, Sheu J, Egger R, De Carvalho TR, Wilson MR, Saha SK, DeRisi JL. Unbiased metagenomic sequencing for Pediatric Meningitis in Bangladesh reveals neuroinvasive Chikungunya Virus Outbreak and other unrealized pathogens. mBio. 2019;10(6):e02877–19. 31848287 10.1128/mBio.02877-19 PMC6918088

[17] Booss J, Esiri MM. Viral encephalitis in humans. Washington, DC: American Society for Microbiology; 2003.

[18] Patricia J, Simner S, Miller, Karen C, Carroll. Understanding the Promises and Hurdles of Metagenomic Next-Generation Sequencing as a Diagnostic Tool for Infectious Diseases, Clinical Infectious Diseases, Volume 66, Issue 5, 1 March 2018, Pages 778–788. 10.1093/cid/cix881 PMC7108102 29040428

[19] Ryan C, Shean, Alexander L, Greninger. One future of clinical metagenomic sequencing for infectious diseases. Expert Rev Mol Diagn. 2019;19(10):849–51. 31426667 10.1080/14737159.2019.1658524

[20] Phillips KA, Douglas MP, Marshall DA. Expanding use of clinical genome sequencing and the need for more data on implementation. JAMA 2020 Nov 24;324(20):2029–30. 10.1001/jama.2020.19933 PMC7686292 33104159

[21] Gilbert GL. Molecular diagnostics in infectious diseases and public health microbiology: cottage industry to postgenomics. Trends Mol Med. 2002;8(6):280–7. 12067614 10.1016/S1471-4914(02)02349-3

[22] Dwivedi S, Purohit P, Misra R, et al. Diseases and Molecular Diagnostics: a step closer to Precision Medicine. Ind J Clin Biochem. 2007;32:374–98. 10.1007/s12291-017-0688-8 PMC5634985 29062170

[23] Krishna NK, Cunnion KM. Role of molecular diagnostics in the management of infectious disease emergencies. Med Clin North Am. 2012;96(6):1067–78. 23102477 10.1016/j.mcna.2012.08.005 PMC7172584

[24] Wilson MR, O’Donovan BD, Gelfand JM, Sample HA, Chow FC, Betjemann JP, Shah MP, Richie MB, Gorman MP, Hajj-Ali RA, Calabrese LH, Zorn KC, Chow ED, Greenlee JE, Blum JH, Green G, Khan LM, Banerji D, Langelier C, Bryson-Cahn C, Harrington W, Lingappa JR, Shanbhag NM, Green AJ, Brew BJ, Soldatos A, Strnad L, Doernberg SB, Jay CA, Douglas V, Josephson SA, DeRisi JL. Chronic meningitis investigated via metagenomic next-generation sequencing. JAMA Neurol. 2018;75:947–55. 29710329 10.1001/jamaneurol.2018.0463 PMC5933460

[25] Langelier C, Kalantar KL, Moazed F, Wilson MR, Crawford E, Deiss T, Belzer A, Bolourchi S, Caldera S, Fung M, Jauregui A, Malcolm K, Lyden A, Khan L, Vessel K, Quan J, Zinter M, Chiu CY, Chow ED, Wilson J, Miller S, Matthay MA, Pollard KS, Christenson S, Calfee CS, DeRisi JL. Integrating host response and unbiased microbe detection for lower respiratory tract infection diagnosis in critically ill adults. Proc Natl Acad Sci USA. 2018;115:E12353–62. 30482864 10.1073/pnas.1809700115 PMC6310811

[26] Wilson MR, Sample HA, Zorn KC, Arevalo S, Yu G, Neuhaus J, Federman S, Stryke D, Briggs B, Langelier C, Berger A, Douglas V, Josephson SA, Chow FC, Fulton BD, DeRisi JL, Gelfand JM, Naccache SN, Bender J, Dien Bard J, Murkey J, Carlson M, Vespa PM, Vijayan T, Allyn PR, Campeau S, Humphries RM, Klausner JD, Ganzon CD, Memar F, Ocampo NA, Zimmermann LL, Cohen SH, Polage CR, DeBiasi RL, Haller B, Dallas R, Maron G, Hayden R, Messacar K, Dominguez SR, Miller S, Chiu CY. Clinical metagenomic sequencing for diagnosis of Meningitis and Encephalitis. N Engl J Med. 2019;380(24):2327–40. 31189036 10.1056/NEJMoa1803396 PMC6764751

[27] Kalantar KL, Carvalho T, de Bourcy CFA, Dimitrov B, Dingle G, Egger R, Han J, Holmes OB, Juan YF, King R, Kislyuk A, Lin MF, Mariano M, Morse T, Reynoso LV, Cruz DR, Sheu J, Tang J, Wang J, Zhang MA, Zhong E, Ahyong V, Lay S, Chea S, Bohl JA, Manning JE, Tato CM, DeRisi JL. IDseq-An open source cloud-based pipeline and analysis service for metagenomic pathogen detection and monitoring. Gigascience. 2020;9(10):giaa111. 33057676 10.1093/gigascience/giaa111 PMC7566497

[28] Namvar L, Olofsson S, Bergström T, Lindh M. Detection and typing of herpes Simplex virus (HSV) in mucocutaneous samples by TaqMan PCR targeting a gB segment homologous for HSV types 1 and 2. J Clin Microbiol. 2005;43(5):2058–64. 15872222 10.1128/JCM.43.5.2058-2064.2005 PMC1153722

[29] Nix WA, Oberste MS, Pallansch MA. Sensitive, seminested PCR amplification of VP1 sequences for direct identification of all enterovirus serotypes from original clinical specimens. J Clin Microbiol. 2006;44(8):2698–704. 16891480 10.1128/JCM.00542-06 PMC1594621

[30] Bachtler M, Frey BM, Frey FJ, Gorgievski M, Simonetti G, Pasch. A Role of enteroviruses in mesangial renal disease [Unpublished].

[31] Quinn KK, Wollersheim SK, Krogstad P. Complete genome sequence of Coxsackievirus B1 isolated during case outbreaks in 2007 in the United States. Genome Announc. 2014;2(4):e00574–14. 10.1128/genomeA.00574-14. PMID: 25059857; PMCID: PMC4110215. 25059857 10.1128/genomeA.00574-14 PMC4110215

[32] Bavelaar HH, Rahamat-Langendoen J, Niesters HG, Zoll J, Melchers WJ. Whole genome sequencing of fecal samples as a tool for the diagnosis and genetic characterization of norovirus. J Clin Virol. 2015;72:122–5. 10.1016/j.jcv.2015.10.003. Epub 2015 Nov 6. PMID: 26492615.]. 26492615 10.1016/j.jcv.2015.10.003

[33] Prasertsopon J, Sangsiriwut K, Noisumdaeng P, Buathong R, Puthavathana P. Enterovirus-associated hand, foot and mouth diseases in Thailand JOURNAL Unpublished. S ubmitted (26-JAN-2022)].

[34] Chehadeh W, Maimoona S, Kurien SS. Dec Molecular characterization of coxsackievirus B1 isolated from 1-year-old child with respiratory distress. J Unpublished Submitted 7 2014.

[35] Zhang T, Du J, Xue Y, Su H, Yang F, Jin Q. Epidemics and frequent recombination within species in outbreaks of Human Enterovirus B-Associated Hand, Foot and Mouth Disease in Shandong China in 2010 and 2011. PLoS ONE. 2013;8(6):e67157. 10.1371/journal.pone.0067157. PMID: 23840610; PMCID: PMC3686723. 23840610 10.1371/journal.pone.0067157 PMC3686723

[36] WHO. Enterovirus Surveillance Guidelines. WHO Regional Office for Europe. 2015. https://www.euro.who.int/__data/assets/pdf_file/0020/272810/EnterovirusSurveillanceGuidelines.pdf.

[37] Rayamajhi, et al. Evaluating cognitive outcomes in adult patients with acute encephalitis syndrome: a prospective study from a tertiary care center in Nepal. Encephalitis. 2022;2(2):36–44. 37469649 10.47936/encephalitis.2021.00157 PMC10295914

[38] Thapa, et al. Clinical profile and outcome of acute encephalitis syndrome (AES) patients treated in College of Medical sciences-Teaching Hospital. J Coll Med Sci. 2013;9(2):31–7.

[39] Rayamajhi A, Ansari I, Ledger E et al. Clinical and prognostic features among children with acute encephalitis syndrome in Nepal; a retrospective study. BMC Infect Dis 11, 294 (2011). 10.1186/1471-2334-11-294 AND. 10.1186/1471-2334-11-294 PMC3219745 22035278

[40] Griffiths MJ, Lemon JV, Rayamajhi A, Poudel P, Shrestha P, Srivastav V et al. (2013) The Functional, Social and Economic Impact of Acute Encephalitis Syndrome in Nepal – a Longitudinal Follow-Up Study. PLoS Negl Trop Dis 7(9): e2383. AND. 10.1371/journal.pntd.0002383 PMC3772013 24069467

[41] Roy DB, Khatri HV. Study of demographic Profile, etiology, and clinical outcome in patients admitted with Acute Encephalitis Syndrome from the western part of India. Cureus. 2022;14(3):e23085. 10.7759/cureus.23085. PMID: 35464588; PMCID: PMC9001832. And. 35464588 10.7759/cureus.23085 PMC9001832

[42] Jmor F, Emsley HC, Fischer M, et al. The incidence of acute encephalitis syndrome in western industrialised and tropical countries. Virol J. 2008;5:134. 10.1186/1743-422X-5-134). 18973679 10.1186/1743-422X-5-134) PMC2583971

[43] Joshi R, Mishra P, Joshi D, et al. Clinical presentation, etiology, and survival in adult acute encephalitis syndrome in rural Central India. Clin Neurol Neurosurg. 2013;115:1753–61. 10.1016/j.clineuro.2013.04.008. 23643180 10.1016/j.clineuro.2013.04.008 PMC3786210

[44] Roy DB, Khatri HV. Study of demographic Profile, etiology, and clinical outcome in patients admitted with Acute Encephalitis Syndrome from the western part of India. Cureus. 2022;14(3):e23085. 10.7759/cureus.23085. PMID: 35464588; PMCID: PMC9001832. 35464588 10.7759/cureus.23085 PMC9001832

[45] Thomas T, Gilbert J, Meyer F. Metagenomics - a guide from sampling to data analysis. Microb Inf Exp. 2012;2(1):3. 10.1186/2042-5783-2-3 PMC3351745 22587947

[46] CZID Portal. h https://chanzuckerberg.zendesk.com/hc/en-us. Accessed July 2022.

[47] Zinter MS, Dvorak CC, Mayday MY, Iwanaga K, Ly NP, McGarry ME, Church GD, Faricy LE, Rowan CM, Hume JR, Steiner ME, Crawford ED, Langelier C, Kalantar K, Chow ED, Miller S, Shimano K, Melton A, Yanik GA, Sapru A, DeRisi JL. Pulmonary metagenomic sequencing suggests missed infections in Immunocompromised Children. Clin Infect Dis. 2019;68(11):1847–55. 30239621 10.1093/cid/ciy802 PMC6784263

[48] Ayling M, Clark MD, Leggett RM. New approaches for metagenome assembly with short reads. Brief Bioinform. 2020;21(2):584–94. 30815668 10.1093/bib/bbz020 PMC7299287

[49] Miller S, Naccache SN, Samayoa E, Messacar K, Arevalo S, Federman S, Stryke D, Pham E, Fung B, Bolosky WJ, Ingebrigtsen D, Lorizio W, Paff SM, Leake JA, Pesano R, DeBiasi R, Dominguez S, Chiu CY. Laboratory validation of a clinical metagenomic sequencing assay for pathogen detection in cerebrospinal fluid. Genome Res. 2019;29:831–42. 30992304 10.1101/gr.238170.118 PMC6499319

[50] Gu W, Crawford ED, O’Donovan BD, Wilson MR, Chow ED, Retallack H, DeRisi JL. Depletion of abundant sequences by hybridization (DASH): using Cas9 to remove unwanted high-abundance species in sequencing libraries and molecular counting applications. Genome Biol. 2016;17:41. 26944702 10.1186/s13059-016-0904-5 PMC4778327

[51] Quan J, Langelier C, Kuchta A, Batson J, Teyssier N, Lyden A, Caldera S, McGeever A, Dimitrov B, King R, Wilheim J, Murphy M, Ares LP, Travisano KA, Sit R, Amato R, Mumbengegwi DR, Smith JL, Bennett A, Gosling R, Mourani PM, Calfee CS, Neff NF, Chow ED, Kim PS, Greenhouse B, DeRisi JL, Crawford ED. FLASH: a next-generation CRISPR diagnostic for multiplexed detection of antimicrobial resistance sequences. Nucleic Acids Res. 2019;47:e83. 31114866 10.1093/nar/gkz418 PMC6698650

[52] Carneiro VCd, Alves-Leon SV, Sarmento DJd, et al. Herpesvirus and neurological manifestations in patients with severe coronavirus disease. Virol J. 2022;19:10. 35676707 10.1186/s12985-022-01828-9 PMC9174631

[53] Patil S, Beck P, Nelson TB, Bran A, Roland W. Herpes simplex Virus-2 Meningoencephalitis with abducens nerve Palsy with Literature Review. Cureus. 2021;13(6):e15523. 34277161 10.7759/cureus.15523 PMC8269988

[54] Kumar A, Shukla D, Kumar R, Idris MZ, Misra UK, Dhole TN. Molecular epidemiological study of enteroviruses associated with encephalitis in children from India. J Clin Microbiol. 2012;50:3509–12. 22895040 10.1128/JCM.01483-12 PMC3486256

[55] Jain S, Patel B, Bhatt GC. Enteroviral encephalitis in children: clinical features, pathophysiology, and treatment advances. Pathog Glob Health. 2014;108(5):216–22. 25175874 10.1179/2047773214Y.0000000145 PMC4153822

[56] Calvo C, Gallardo P, Torija P, Bellón S, Méndez-Echeverría A, Del Rosal T, Baquero-Artigao F, Sainz T, Romero M, Cabrerizo M. Enterovirus neurological disease and bacterial coinfection in very young infants with fever. J Clin Virol. 2016;85:37–9. 27833059 10.1016/j.jcv.2016.10.020

[57] Genus. Enterovirus. International Committee on Taxonomy of Viruses (ICTV). Accessed: 18 July 2022. Derivation of names Entero: from Greek enteron, ‘intestine’.

[58] Chaudhary MC, Bronze MS. Enterovirus Infection. Medscape: Infectious Diseases. 2019. Accessed: 18 July 2022.

[59] Glaser CA, Gilliam S, Schnurr D, et al. In search of encephalitis etiologies: diagnostic challenges in the California Encephalitis Project, 1998–2000. Clin Infect Dis. 2003;36(6):731–42. 12627357 10.1086/367841

[60] Gofshteyn J, Cárdenas AM, Bearden D. Treatment of chronic enterovirus encephalitis with fluoxetine in a patient with X-linked agammaglobulinemia. Pediatr Neurol. 2016;64:94–8. 27640319 10.1016/j.pediatrneurol.2016.06.014

[61] Wikswo ME, Khetsuriani N, Fowlkes AL, Zheng X, Peñaranda S, Verma N, Shulman ST, Sircar K, Robinson CC, Schmidt T, Schnurr D, Oberste MS. Increased activity of Coxsackievirus B1 strains associated with severe disease among young infants in the United States, 2007–2008. Clin Infect Dis. 2009;49(5):e44-51. 10.1086/605090. PMID: 19622041. 10.1086/605090 19622041

[62] Sapkal GN, Sapkal GN, Bondre VP, Fulmali PV. Enteroviruses in patients with acute encephalitis, Uttar pradesh, India. Emerg Infect Dis. 2009;15:295–8. 19193277 10.3201/eid1502.080865 PMC2657625

[63] Mittal M, Kushwaha KP, Pandey AK, Fore MM. A clinico-epidemiological study of acute encephalitis syndrome with multi organ dysfunction. Int J Contemp Pediatr. 2017. 4(3).

[64] Ravi V, Hameed SKS, Desai A, Mani RS, Reddy V, Velayudhan A, Yadav R, Jain A, Saikia L, Borthakur AK, Sharma A, Mohan DG, Bhandopadhyay B, Bhattacharya N, Inamdar L, Hossain S, Daves S, Sejvar J, Dhariwal AC, Sen PK, Venkatesh S, Prasad J, Laserson K, Srikantiah P. An algorithmic approach to identifying the aetiology of acute encephalitis syndrome in India: results of a 4-year enhanced surveillance study. Lancet Glob Health. 2022;10(5):e685–93. 35427525 10.1016/S2214-109X(22)00079-1

[65] Mittal M, Bondre V, Murhekar M, Deval H, Rose W, Verghese VP, Mittal M, Patil G, Sabarinathan R, Vivian Thangaraj JW, Kanagasabai K, Prakash JAJ, Gupta N, Gupte MM, Gupte MD. Acute Encephalitis Syndrome in Gorakhpur, Uttar Pradesh, 2016: Clinical and Laboratory findings. Pediatr Infect Dis J. 2018;37(11):1101–6. 29746378 10.1097/INF.0000000000002099

[66] Kumar A, Shukla D, Kumar R, Idris MZ, Misra UK, Dhole TN. An epidemic of encephalitis associated with human enterovirus B in Uttar Pradesh, India, 2008. J Clin Virol. 2011;51(2):142–5. 21444241 10.1016/j.jcv.2011.02.011

[67] Giri A, Arjyal A, Koirala S, Karkey A, Dongol S, Thapa SD, Shilpakar O, Shrestha R, van Tan L, Thi Thuy Chinh BN, Krishna KCR, Pathak KR, Shakya M, Farrar J, Van Doorn HR, Basnyat B. Aetiologies of central nervous system infections in adults in Kathmandu, Nepal: a prospective hospital-based study. Sci Rep. 2013;3:2382. 23924886 10.1038/srep02382 PMC3737500

[68] Säll O, Thulin Hedberg S, Neander M, Tiwari S, Dornon L, Bom R, Lagerqvist N, Sundqvist M, Mölling P. Etiology of Central Nervous System infections in a rural area of Nepal using Molecular approaches. Am J Trop Med Hyg. 2019;101(1):253–9. 31162021 10.4269/ajtmh.18-0434 PMC6609203

[69] Bookstaver PB, Mohorn PL, Shah A, Tesh LD, Quidley AM, Kothari R, Bland CM, Weissman S. Management of viral central nervous system infections: a primer for clinicians. J Cent Nerv Syst Dis. 2017. 9. 10.1177/1179573517703342 PMC5415352 28579869

[70] AK AK, Mendez MD, Herpes Simplex E. 2022 Mar 15. In: StatPearls [Internet]. Treasure Island (FL): StatPearls Publishing; 2022 Jan–. PMID: 32491575.

[71] Khetsuriani N, Holman RC, Anderson LJ. Burden of encephalitis-associated hospitalizations in the United States, 1988–1997. Clinical infectious diseases: an official publication of the Infectious Diseases Society of America. 2002. 35. 175–82. 10.1086/341301 12087524

[72] Davison KL, Crowcroft NS, Ramsay ME, Brown DW, Andrews NJ. Viral encephalitis in England, 1989–1998: what did we miss? Emerging infectious diseases. 2003. 9. 234–240. 10.3201/eid0902.020218 PMC2901942 12603996

[73] Huppatz C, et al. Etiology of encephalitis in Australia, 1990–2007. Emerg Infect Dis. 2009;15:1359–65. 19788802 10.3201/eid1509.081540 PMC2819877

[74] Panagariya A, Jain RS, Gupta S, Garg A, Sureka RK, Mathur V. Herpes simplex encephalitis in North West India. Neurol India. 2001;49(4):360–5. 11799408

[75] Bradshaw MJ, Venkatesan A. Herpes simplex Virus-1 encephalitis in adults: pathophysiology, diagnosis, and management. Neurotherapeutics. 2016;13(3):493–508. 27106239 10.1007/s13311-016-0433-7 PMC4965403

[76] Kubo T, Rai SK, Nakanishi M, Yamano T. Seroepidemiological study of herpes viruses in Nepal. Southeast Asian J Trop Med Public Health. 1991;22(3):323–5. 1667956

[77] Adhikari A. A case report on herpes Simplex Encephalitis. Med J Shree Birendra Hosp. 2012. 11(1).

[78] Rayamajhi P, Nepal G, Ojha R, Rajbhandari R, Gajurel BP, Karn R. Evaluating cognitive outcomes in adult patients with acute encephalitis syndrome: a prospective study from a tertiary care center in Nepal. 2022. 2(2). 36–44. 10.47936/encephalitis.2021.00157 PMC10295914 37469649

[79] Ramesh A, Nakielny S, Hsu J, Kyohere M, Byaruhanga O, de Bourcy C, Egger R, Dimitrov B, Juan YF, Sheu J, Wang J, Kalantar K, Langelier C, Ruel T, Mpimbaza A, Wilson MR, Rosenthal PJ, DeRisi JL. Metagenomic next-generation sequencing of samples from pediatric febrile illness in Tororo, Uganda. PLoS ONE. 2019;14(6):e0218318. 31220115 10.1371/journal.pone.0218318 PMC6586300

[80] Institute of Medicine (US) Roundtable on Value & Science-Driven Health Care. Clinical Data as the Basic Staple of Health Learning: Creating and Protecting a Public Good: Workshop Summary. Washington (DC): National Academies Press (US). 2010. https://www.ncbi.nlm.nih.gov/books/NBK54302/. 21595112

[81] Leys NM, Ryngaert A, Bastiaens L, Verstraete W, Top EM, Springael D. Occurrence and phylogenetic diversity of Sphingomonas strains in soils contaminated with polycyclic aromatic hydrocarbons. Appl Environ Microbiol. 2004;70(4):1944–55. 15066784 10.1128/AEM.70.4.1944-1955.2004 PMC383131

[82] Cavicchioli R, Fegatella F, Ostrowski M, Eguchi M, Gottschal J. Sphingomonads from marine environments. J Ind Microbiol Biotechnol. 1999;23(4–5):268–72. 11423943 10.1038/sj.jim.2900732

[83] Thraenhart O, Jursch C. Virucidal activity if the nucleic acid preservation product DNA/RNA Shield against the murine parvovirus (MVM) at 20°C. Eurovir; 2018.

[84] Phommanivong V, Kanda S, Shimono T, et al. Co-circulation of the dengue with Chikungunya virus during the 2013 outbreak in the southern part of Lao PDR. Trop Med Health. 2016;44:24. 27524929 10.1186/s41182-016-0020-y PMC4973078

[85] New York State Department of Health. Collection and Submission of Specimens for Viral Encephalitis Testing. July 2010.

[86] iSeq100 Specifications. Accessed: 18 July 2022. https://support.illumina.com/bulletins/2020/04/maximum-read-length-for-illumina-sequencing-platforms.html.

